# Uric acid shown to contribute to increased oxidative stress level independent of xanthine oxidoreductase activity in MedCity21 health examination registry

**DOI:** 10.1038/s41598-021-86962-0

**Published:** 2021-04-01

**Authors:** Masafumi Kurajoh, Shinya Fukumoto, Shio Yoshida, Seigo Akari, Takayo Murase, Takashi Nakamura, Haruka Ishii, Hisako Yoshida, Yuki Nagata, Tomoaki Morioka, Katsuhito Mori, Yasuo Imanishi, Kazuto Hirata, Masanori Emoto

**Affiliations:** 1 Department of Metabolism, Endocrinology, and Molecular Medicine, Osaka City University Graduate School of Medicine, 1-4-3, Asahi-machi, Abeno-ku, Osaka, 545-8585 Japan; 2 Department of Premier Preventive Medicine, Osaka City University Graduate School of Medicine, Osaka, Japan; 3 Department of Research and Development, Sanwa Kagaku Kenkyusho Co., Ltd., Aichi, Japan; 4 Department of Medical Statistics, Osaka City University Graduate School of Medicine, Osaka, Japan; 5 Department of Vascular Medicine, Osaka City University Graduate School of Medicine, Osaka, Japan; 6 Department of Nephrology, Osaka City University Graduate School of Medicine, Osaka, Japan; 7 Osaka City University, Osaka, Japan

**Keywords:** Metabolic diseases, Metabolic syndrome

## Abstract

Uric acid has both antioxidant and pro-oxidant properties in vitro by scavenging and production of reactive oxygen species (ROS)*.* This cross-sectional study examined whether uric acid possesses effects on oxidative stress under physiological conditions independent of xanthine oxidoreductase (XOR), which is involved in uric acid and ROS production. Serum uric acid level was measured, while plasma XOR activity was determined using our high-sensitive assay in 192 participants (91 males, 101 females) who underwent health examinations and were not taking an antihyperuricemic agent. For antioxidant potential and oxidative stress level, biological antioxidant potential (BAP) and derivative of reactive oxygen metabolites (d-ROMs) in serum, respectively, were measured. Median uric acid level and plasma XOR activity were 5.6 mg/dL and 26.1 pmol/h/mL, respectively, and BAP and d-ROMs levels were 2112.8 μmol/L and 305.5 Carr U, respectively. Multivariable regression analyses revealed no significant association of serum uric acid level with BAP level, whereas serum uric acid level showed a significant association with d-ROMs level independent of plasma XOR activity (*p* = 0.045), which was prominent in females (*p* = 0.036; *p* for interaction = 0.148). Uric acid might contribute to increased oxidative stress independent of XOR activity by increasing ROS production, without affecting ROS scavenging, especially in females.

## Introduction

Uric acid is produced by a xanthine oxidoreductase (XOR)-catalyzed reaction, and shown to have both antioxidant and pro-oxidant properties in vitro by scavenging and production of reactive oxygen species (ROS)^1,2^. However, its role in regulating oxidative stress under physiological conditions remains unclear, as such a reaction produces ROS as well as uric acid^3,4^.

Although several methods to determine circulating XOR activity have been reported, such as with use of an ultraviolet (UV) detector or liquid chromatography (LC)/UV^5,6^, there are difficulties with obtaining accurate measurements of circulating XOR activity in humans, as it is extremely lower than that in rodents^7^. We recently developed a highly sensitive test for human plasma XOR activity that utilizes assay results of stable isotope-labeled [^13^C_2_,^15^N_2_] xanthine obtained with LC/triple quadrupole mass spectrometry (TQMS)^8,9^. Using this method, associations of plasma XOR activity with serum uric acid level have been found in both cross-sectional and longitudinal studies^10–12^, suggesting that XOR activity in plasma reflects systemic XOR activity.

Some previous studies conducted in clinical settings have reported an association between uric acid and oxidative stress level^13–15^. However, to the best of our knowledge, no investigation of the association of uric acid with oxidative stress level adjustment for XOR activity in humans has been reported. To determine whether uric acid has an effect on oxidative stress level independent of XOR activity under physiological conditions, the associations of serum uric acid level and plasma XOR activity with levels of reactive biological antioxidant potential (BAP) and oxygen metabolites (d-ROMs), markers of antioxidant potential and oxidative stress, respectively^16^, were examined using our novel XOR activity assay in subjects who voluntarily underwent a health examination.

## Results

### Clinical characteristics of subjects

Characteristics of the enrolled subjects are shown in Table 1. The median values for uric acid and plasma XOR activity were 5.6 mg/dL and 26.1 pmol/h/mL, respectively, while those for BAP and d-ROMs level were 2112.8 μmol/L and 305.5 Carr U, respectively.

**Table 1:** Clinical characteristics of subjects (n = 192).

Age, years	56.0 (47.0–67.0)
Males, n	91 (47.4)
Smoking habit, n	39 (20.3)
Alcohol habit, n	101 (52.6)
Body mass index, kg/m^2^	22.6 (20.7–24.7)
VFA, cm^2^	69.7 (43.4–106.9)
SBP, mmHg	121.0 (110.0–132.0)
DBP, mmHg	74.0 (66.0–82.0)
Total cholesterol, mg/dL	204.0 (181.0–225.3)
Triglyceride, mg/dL	89.0 (62.8–127.3)
HDL cholesterol, mg/dL	60.0 (50.8–70.0)
FPG, mg/dL	100.0 (94.8–108.3)
HbA1c, %	5.7 (5.5–6.0)
eGFR, mL/min/1.73 m^2^	76.8 (66.2–86.5)
HOMA-IR	1.4 (0.9–2.1)
hs-CRP, ng/mL	328.0 (146.0–698.8)
Uric acid, mg/dL	5.6 (4.4–6.4)
Plasma XOR activity, pmol/h/mL	26.1 (15.4–57.3)
BAP, µmol/L	2112.8 (2013.1–2233.2)
d-ROMs, CarrU	305.5 (276.8–343.3)

Data are expressed as the median (inter-quartile range) or number (%).

*VFA* visceral fat area, *SBP* systolic blood pressure, *DBP* diastolic blood pressure, *HDL* high-density lipoprotein, *FPG* fasting plasma glucose, *HbA1c* glycated hemoglobin, *eGFR* estimated glomerular filtration rate, *HOMA-IR* homeostatic model assessment of insulin resistance, *hs-CRP* high-sensitivity C-reactive protein, *XOR* xanthine oxidoreductase, *BAP* biological antioxidant potential, *d-ROMs* derivative of reactive oxygen metabolites.

### No association of serum uric acid level with BAP level

To examine whether uric acid level was independently associated with BAP level after adjustment for other confounding factors including plasma XOR activity, multivariable regression analyses were performed (Table 2). While Visceral fat area (VFA) and total cholesterol were significantly associated (*p* < 0.05), neither serum uric acid level nor plasma XOR activity showed a significant association with BAP level.

**Table 2:** Multivariable regression analysis of possible factors associated with BAP level.

Independent variable	Percentile		Coefficient	*p* value
	25th	75th	Difference (95% CI)	
Age	47.0	67.0	1.519 (− 51.251, 54.289)	0.955
Gender (male = 1, female = 0)	0	1	− 25.630 (− 89.692, 38.431)	0.431
Smoking habit (present = 1, absent = 0)	0	1	− 34.585 (− 98.650, 29.480)	0.288
Alcohol drinking habit (present = 1, absent = 0)	0	1	− 23.585 (− 77.081, 29.911)	0.385
VFA	43.4	106.9	− 55.764 (− 108.527, − 3.001)	0.038
SBP	110.0	132.0	4.042 (− 35.146, 43.230)	0.839
Total cholesterol	181.0	225.3	− 68.697 (− 103.793, − 33.601)	< 0.001
HbA1c	5.5	6.0	16.644 (− 15.704, 48.991)	0.311
eGFR	66.2	86.5	− 30.898 (− 75.673, 13.878)	0.175
Log HOMA-IR	− 0.046	0.322	− 4.802 (− 45.697, 36.092)	0.817
Log hs-CRP	2.164	2.844	5.187 (− 33.136, 43.510)	0.790
Uric acid	4.4	6.4	29.840 (− 25.757, 85.436)	0.381
Log XOR	1.186	1.758	− 15.097 (− 58.010, 27.817)	0.488

*BAP* biological antioxidant potential, *VFA* visceral fat area, *SBP* systolic blood pressure, *HbA1c* glycated hemoglobin, *eGFR* estimated glomerular filtration rate, *HOMA-IR* homeostatic model assessment of insulin resistance, *hs-CRP* high-sensitivity C-reactive protein, *XOR* xanthine oxidoreductase, *CI* confidence interval.

### Association between uric acid and d-ROMs levels in serum is independent of plasma XOR activity

**Figure 1:**
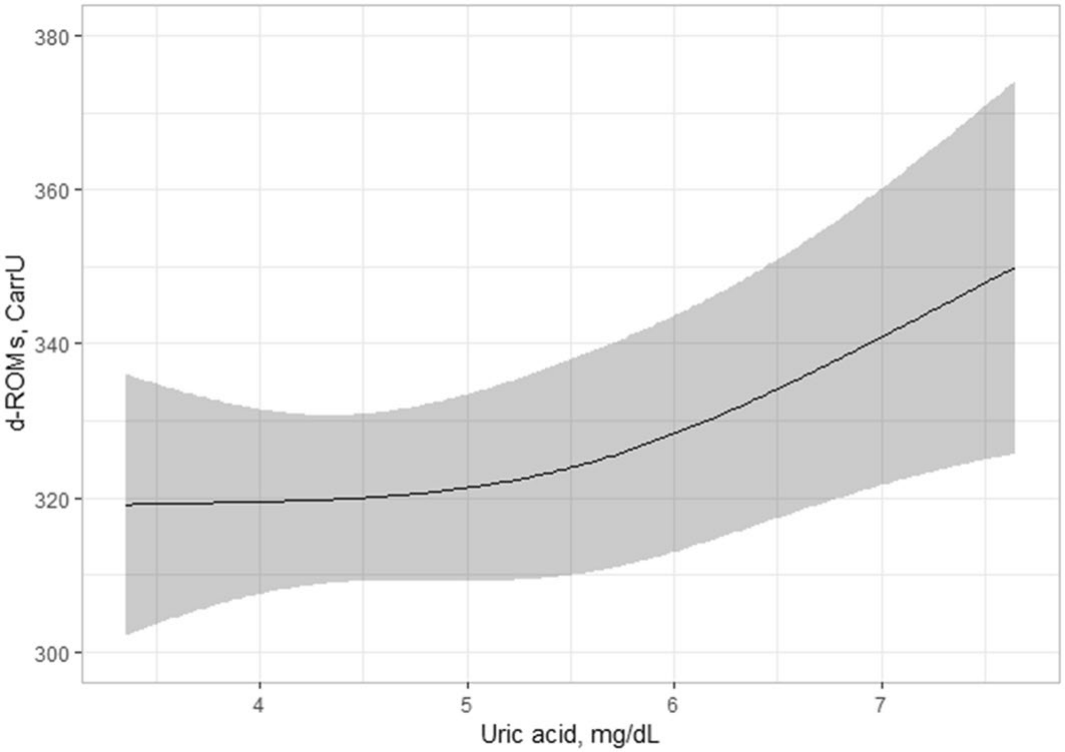
Serum uric acid and d-ROMs levels. The serum level of uric acid was significantly associated with d-ROMs level (*p* = 0.045) and the nonlinear effect of that on d-ROMs level was also significant (*p* = 0.171). Best fitted line and 95% CI results are shown by a solid line and gray band, respectively. Values for d-ROMs were adjusted according to the median values for age, gender, smoking habit, alcohol drinking habit, VFA, SBP, total cholesterol, HbA1c, eGFR, log HOMA-IR, log hs-CRP, and log plasma XOR activity. *d-ROMs* derivative of reactive oxygen metabolites, *CI* confidence interval, *VFA* visceral fat area, *SBP* systolic blood pressure, *HbA1c* glycated hemoglobin, *eGFR* estimated glomerular filtration rate, *HOMA-IR* homeostatic model assessment of insulin resistance, *hs-CRP* high-sensitivity C-reactive protein, *XOR* xanthine oxidoreductase.

**Figure 2:**
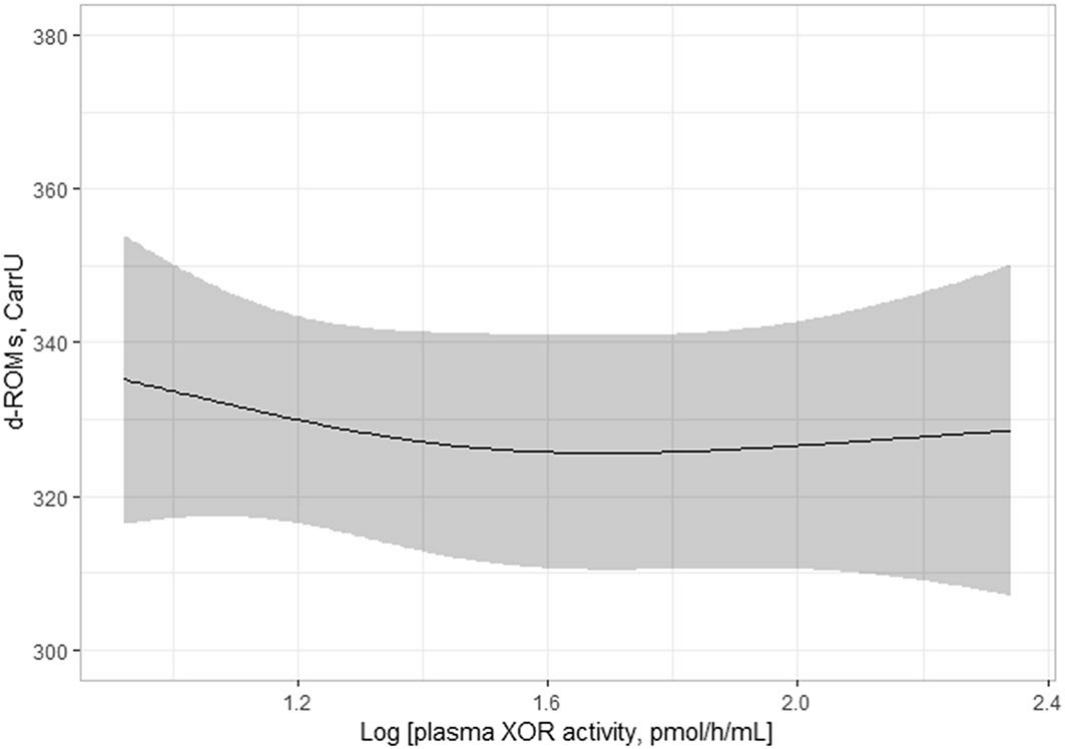
Plasma XOR activity and d-ROMs level. Plasma XOR activity was not significantly associated with d-ROMs level (*p* = 0.725). Best fitted line and 95% CI results are shown by a solid line and gray band, respectively. Values for d-ROMs were adjusted according to the median values for age, gender, smoking habit, alcohol drinking habit, VFA, SBP, total cholesterol, HbA1c, eGFR, log HOMA-IR, log hs-CRP, and uric acid level. *XOR* xanthine oxidoreductase, *d-ROMs* derivative of reactive oxygen metabolites, *CI* confidence interval, *VFA* visceral fat area, *SBP* systolic blood pressure, *HbA1c* glycated hemoglobin, *eGFR* estimated glomerular filtration rate, *HOMA-IR* homeostatic model assessment of insulin resistance, *hs-CRP* high-sensitivity C-reactive protein.

To examine whether serum uric acid level was independently associated with d-ROMs level after adjustment for other confounding factors including plasma XOR activity, multivariable regression analyses were performed (Table 3). Uric acid level, but not plasma XOR activity, was significantly associated with d-ROMs level (*p* = 0.045) (Figs. 1, 2), as were gender as well as VFA and high-sensitivity C-reactive protein (hs-CRP) (*p* < 0.05). On the other hand, no such association was seen with age, smoking habit, alcohol drinking habit, systolic blood pressure (SBP), total cholesterol, glycated hemoglobin (HbA1c), estimated glomerular filtration rate (eGFR), or homeostatic model assessment of insulin resistance (HOMA-IR).

**Table 3:** Multivariable regression analysis of possible factors associated with d-ROMs level.

Independent variable	Percentile		Coefficient	*p* value
	25th	75th	Difference (95% CI)	
Age	47.0	67.0	13.234 (− 0.506, 26.974)	0.059
Gender (male = 1, female = 0)	0	1	− 52.461 (− 69.141, − 35.780)	< 0.001
Smoking habit (present = 1, absent = 0)	0	1	14.547 (− 2.134, 31.228)	0.087
Alcohol drinking habit (present = 1, absent = 0)	0	1	8.476 (− 5.453, 22.406)	0.231
VFA	43.4	106.9	− 18.946 (− 32.685, − 5.208)	0.007
SBP	110.0	132.0	− 2.913 (− 13.117, 7.291)	0.574
Total cholesterol	181.0	225.3	6.883 (− 2.255, 16.021)	0.139
HbA1c	5.5	6.0	0.321 (− 8.102, 8.744)	0.940
eGFR	66.2	86.5	1.850 (− 9.808, 13.509)	0.754
Log HOMA-IR	− 0.046	0.322	− 1.408 (− 12.056, 9.240)	0.794
Log hs-CRP	2.164	2.844	30.355 (20.376, 40.333)	< 0.001
Uric acid	4.4	6.4	Non-linear effect^a^	0.045
Log XOR	1.186	1.758	− 1.991 (− 13.165, 9.183)	0.725

*d-ROMs* derivative of reactive oxygen metabolites, *VFA* visceral fat area, *SBP* systolic blood pressure, *HbA1c* glycated hemoglobin, *eGFR* estimated glomerular filtration rate, *HOMA-IR* homeostatic model assessment of insulin resistance, *hs-CRP* high-sensitivity C-reactive protein, *XOR* xanthine oxidoreductase, *CI* confidence interval.

^a^See Fig. 1.

### Association of serum uric acid level with d-ROMs level stratified by gender

**Figure 3:**
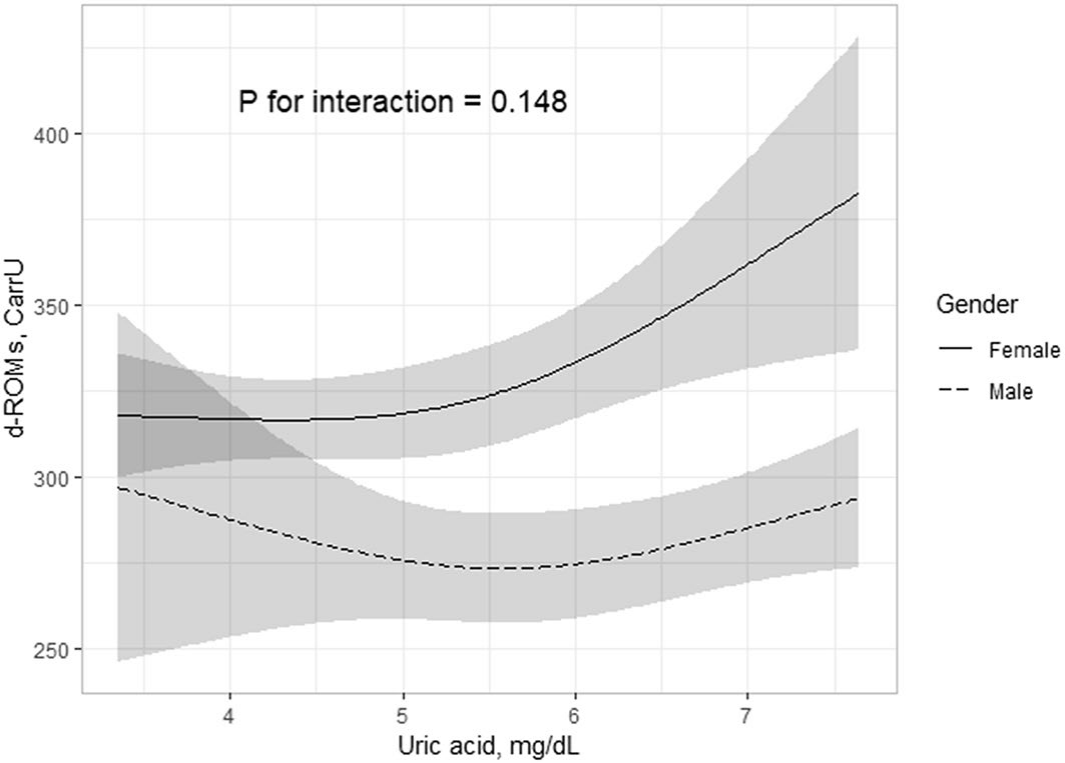
Serum uric acid and d-ROMs levels stratified by gender. The serum level of uric acid was significantly associated with d-ROMs level in females (*p* = 0.036) but not in males (*p* = 0.115). The nonlinear effect of serum uric acid level on d-ROMs level was significant (*p* = 0.066). Best fitted line and 95% CI results are shown by a solid line and dark gray band, respectively. Values for d-ROMs were adjusted according to the median values for age, smoking habit, alcohol drinking habit, VFA, SBP, total cholesterol, HbA1c, eGFR, log HOMA-IR, log hs-CRP, and log plasma XOR activity after stratification by gender. *d-ROMs* derivative of reactive oxygen metabolites, *CI* confidence interval, *VFA* visceral fat area, *SBP* systolic blood pressure, *HbA1c* glycated hemoglobin, *eGFR* estimated glomerular filtration rate, *HOMA-IR* homeostatic model assessment of insulin resistance, *hs-CRP* high-sensitivity C-reactive protein, *XOR* xanthine oxidoreductase.

To further examine whether the association of uric acid level with d-ROMs level was affected by gender, interaction analyses were performed. The “gender * uric acid” interaction was significant (*p* = 0.148), while uric acid level was shown to be significantly associated with d-ROMs level in females (*p* = 0.036) but not males (*p* = 0.115) (Fig. 3), suggesting that gender has an effect on the relationship between uric acid and d-ROMs in serum. On the other hand, plasma XOR activity showed no significant association with d-ROMs level in either females (*p* = 0.350) or males (*p* = 0.394).

## Discussion

This is the first known study to investigate the relationship of uric acid level and XOR activity with oxidative stress level in humans. In analyses of subjects who voluntarily underwent a health examination, our findings showed that the level of uric acid in serum had no significant association with BAP level independent of plasma XOR activity (Table 2). In contrast, serum uric acid level showed a significant association with d-ROMs level independent of plasma XOR activity (Table 3; Figs. 1, 2). Furthermore, the significant association of serum uric acid level with d-ROMs level was more prominent in females as compared to males (Fig. 3). Together, these results suggest that uric acid contributes to an increase in oxidative stress level by increasing ROS production under physiological conditions in a manner independent of XOR activity, especially in females. On the other hand, the antioxidant effects of uric acid under physiological conditions might be counterbalanced by ROS produced by an XOR-catalyzed reaction.

Oxidative stress is caused by a disturbance of the relationship between antioxidant activity and ROS production, and several markers have been proposed for evaluation of oxidative stress and antioxidant activity. Tests for BAP and d-ROMs, markers of antioxidant activity and oxidative stress, respectively, have been experimentally validated by results obtained with electron spin resonance, the gold standard of techniques actually available for free radical studies^16,17^. Furthermore, BAP testing has been shown to provide results comparable to other markers of antioxidant capacity, such as ferric-reducing ability and plasma antioxidant tests^18,19^, while a d-ROMs test was also demonstrated to provide results comparable to other markers of oxidative stress, such as FOX assay and 8-isoprostane^20,21^. Therefore, in the present study, we used BAP and d-ROMs tests to investigate the association of uric acid and XOR activity with antioxidant capacity and oxidative stress level.

In in vitro findings, uric acid has been shown to gain antioxidant properties by scavenging ROS, such as singlet oxygen, peroxyl radical, hydroxyl radical, and peroxynitrite^1,22^. Furthermore, serum uric acid levels were reported to be positively associated with markers of antioxidant potential in subjects who underwent a health examination^14,15,23^. However, those associations were not analyzed after adjustment for the activity of XOR, which produces ROS in addition to uric acid^3,4^. In the present results of multivariable regression analysis including plasma XOR activity as a covariate, uric acid was not significantly associated with BAP level (Table 2). Of interest, the antioxidant effects of uric acid in humans have been reported in examinations performed under non-physiological settings such as following exogeneous administration. For example, administration of uric acid or its precursor inosine improved endothelial function in patients with type 1 diabetes^24^, as well as clinical functional outcomes in patients with acute ischemic stroke^25^ or multiple sclerosis^26^. On the other hand, hyperuricemia was shown to be associated with endothelial dysfunction^27,28^ and used to predict poor clinical functional outcomes in patients with acute ischemic stroke^29^. Thus, the present results along with those in previous studies suggest that uric acid exerts antioxidant effects mainly under non-physiological conditions, such as exogeneous administration, while those effects are counterbalanced by ROS produced by an XOR-catalyzed reaction under physiological conditions.

In addition to antioxidant properties, uric acid is known to have pro-oxidant properties by generating ROS such as superoxide anions, an activity that is mediated by activation of nicotinamide adenine dinucleotide phosphate oxidase in adipocytes, as well as vascular smooth muscle and endothelial cells^2,30,31^. Consistent with previous in vitro findings, serum uric acid levels were reported to be positively associated with markers of oxidative stress levels in clinical settings^13–15^. However, such associations might actually reflect an association of XOR-derived ROS with oxidative stress level, since ROS and also uric acid are produced by an XOR-catalyzed reaction^3,4^, and plasma XOR activity has been shown to be positively associated with serum uric acid level^10–12^. In the present study, serum uric acid level, but not plasma XOR activity, showed a significant association with d-ROMs level (Table 3; Figs. 1, 2), suggesting that uric acid contributes to increased oxidative stress level independent of XOR activity through increased ROS production.

The association of serum uric acid level with d-ROMs level was prominent in females as compared to males in the present study (Fig. 3). Those results are consistent with previous reports showing that the association of serum uric acid level with oxidative stress level was more prominent in their female subjects^13,14^. Oxidative stress is considered to have a major role in the pathogenesis of lifestyle-related conditions, such as hypertension, nonalcoholic fatty liver disease, and metabolic syndrome, and also cardiovascular and cerebrovascular diseases^32–35^. On the other hand, previous meta-analysis findings showed that females with hyperuricemia had increased risk for development of the above-mentioned lifestyle-related diseases as compared to males with hyperuricemia^36–40^. Thus, we speculate that uric acid might be involved in the pathophysiology of lifestyle-related diseases by an association with increased oxidative stress, especially in females. Nevertheless, the underlying mechanisms involved in the gender-specific association of uric acid level in serum with oxidative stress level require further investigation.

The present study has several limitations. First, none of the enrolled subjects had hypouricemia, defined as a uric acid level in serum ≤ 2.0 mg/dL^41^, and few had hyperuricemia (n = 24), shown by a serum uric acid level > 7.0 mg/dL, as they were sequentially selected from individuals who voluntarily participated in a health examination. Although no significant association was noted between serum uric acid and BAP levels, there was a significant J-curve (nonlinear) association between serum uric acid and d-ROMs levels. However, we were not able to analyze those associations with stratification by hypouricemia or hyperuricemia. In addition, the association between serum uric acid and d-ROMs level seems to show a J-curve trendline at lower levels of serum uric acid, especially in males (Fig. 3). However, we were not able to analyze those associations by stratification with a low- or high-normal serum uric acid level, due to the low number of male subjects with a low-normal serum uric acid level. Second, the number of subjects with chronic kidney disease (CKD), defined as eGFR < 60 mL/min/1.73 m^2^, was few (n = 22) and none of the enrolled subjects had end-stage renal disease (ESRD), defined as eGFR < 15 mL/min/1.73 m^2^, or need for dialysis or renal transplantation. Although we previously found a positive association of plasma XOR activity with serum uric acid level in subjects who underwent health examinations as well as patients receiving hemodialysis treatments^10–12^, an inverse association of renal function with serum uric acid level independent of plasma XOR activity was also been revealed in our other results^11,12^, suggesting that the balance of serum uric acid level and plasma XOR activity might be different between individuals with and without CKD/ESRD. Third, we did not measure levels of antioxidants, such as vitamin C, glutathione, vitamin E, and polyphenol, thus were not able to comprehensively investigate the association between uric acid and antioxidant capacity including antioxidant levels. Fourth, uric acid administration testing was not performed, as the aim of the present study was to determine whether uric acid has an effect on oxidative stress level independent of XOR activity under physiological conditions. Therefore, differences in antioxidant and/or pro-oxidant effects exerted by intrinsic and exogenous uric acid were not clarified. Finally, because of the cross-sectional design, even though relationships were explored in predictive terms, the results cannot be interpreted to show causal relationships. A large-scale longitudinal study that includes subjects with hypouricemia, low-normal uricemia, hyperuricemia, or CKD/ESRD, as well as measurements of antioxidant levels and uric acid administration testing is needed to clarify the role of uric acid in regulation of oxidative stress.

In conclusion, the level of uric acid in serum showed a significant association with d-ROMs level in serum independent of plasma XOR activity in subjects registered in the MedCity21 health examination registry. That association was found to be more prominent in females, while there was no independent association with BAP level. These results suggest that uric acid contributes to increased oxidative stress by causing an imbalance between XOR-independent ROS generation and XOR-counterbalanced ROS scavenging, especially in females.

## Subjects and methods

### Study design

The MedCity21 health examination registry was initiated in April 2015 in a comprehensive manner to elucidate causes of various diseases occurring in adults, including cancer, diabetes mellitus, cardiovascular disease, cerebrovascular disease, mental disorders, dyslipidemia, hypertension, hyperuricemia, obesity, chronic respiratory disease, liver disease, digestive disease, gynecological diseases, and skin disease, for development of advanced diagnostic techniques, as well as treatment and prevention methods for affected individuals^11,42,43^. Those who voluntarily underwent heath examinations at MedCity21, an advanced medical center for preventive medicine established at Osaka City University Hospital (Osaka, Japan), were registered. The MedCity21 health examination registry protocol has been approved by the Ethics Committee of Osaka City University Graduate School of Medicine (Approval No. 2927). Written informed consent was obtained from all subjects and the study was conducted in full accordance with the Declaration of Helsinki. The present study protocol was approved by the Ethics Committee of Osaka City University Graduate School of Medicine (Approval No. 3684) and performed with an opt-out option, as explained in instructions posted on the website of the hospital.

### Participants

Referring to the MedCity21 health examination registry of individuals examined between June 2015 and May 2017, the final 200 sequential subjects who participated in advanced comprehensive medical examinations designed to check the status of lifestyle-related diseases, such as hypertension, diabetes, dyslipidemia, visceral obesity, hyperuricemia, atherosclerosis, and cerebrovascular disease, were selected. For the present analysis, those being treated with an XOR inhibitor (n = 4), or uricosuric (n = 1) or insulin (n = 1) agents, or with missing data (n = 2) were excluded. As a result, 192 participants (91 males, 101 females) were enrolled as subjects in the present cross-sectional study.

### Clinical assessments

Information for each subject regarding height, body weight, SBP, smoking and alcohol consumption habits, present and past illness, and use of medication was obtained. Body mass index was calculated as weight in kilograms divided by the square of height in meters (kg/m^2^). VFA values were obtained using a computed tomography device (Supria Grande, Hitachi, Ltd., Tokyo, Japan), as previously described^44,45^. Blood was drawn after an overnight fast, then biochemical parameters including serum uric acid level were analyzed using a standard laboratory method as part of the MedCity21 protocol and remaining blood samples were stored at − 80 °C. HbA1c level was determined as a National Glycohemoglobin Standardization Program equivalent value (%) using the conversion formula established by the Japan Diabetes Society^46^. eGFR was calculated using an equation designed for Japanese subjects, as previously described^47^. Serum immunoreactive insulin (IRI) level was measured with an electrochemiluminescence immunoassay (Roche Diagnostics K.K., Tokyo, Japan). The HOMA-IR index was calculated according to the following formula: fasting IRI (lU/mL) × fasting plasma glucose (mg/dL)/405^48,49^. The serum concentration of hs-CRP was measured using latex agglutination nephelometry (Siemens Healthcare Diagnostics, Inc., Marburg, Germany), as previously described^50^.

### Serum BAP and d-ROMs levels

Freshly frozen serum samples were maintained at − 80 °C until the time of the assay. Levels of BAP and d-ROMs were assessed as markers of antioxidant potential and oxidative stress level, respectively, using a free radical elective evaluator system (FREE: Diacron International s.r.l., Grosseto, Italy) that included a spectrophotometric device reader, as previously described^16,44,51^. Briefly, for BAP, 50 μL of a ferric chloride solution was mixed with a thiocyanate derivative solution in a cuvette. Subsequently, 10 μL of serum was added and mixed, which resulted in a reduction of iron contained therein from a ferric to ferrous form by the actions of antioxidants. Color change in the cuvette was determined using a spectrophotometer at a wavelength of 505 nm and expressed as μmol/L. The intra- and inter-assay coefficients of variation in BAP level were 2.2% and 3.1%, respectively. As for d-ROMs testing, 20 μL of serum was mixed with an acid buffer solution (pH 4.8) in a cuvette. Subsequently, a chromogen for d-ROMS (reagent containing, N,N-diethyl-p-phenylenediamine, 20-μL) was added and mixed, resulting in oxidation of the chromogen substrate by free radicals. Color change in the cuvette was determined using a spectrophotometer at a wavelength of 505 nm and expressed as Carr units. The intra- and inter-assay coefficients of variation in d-ROMs level were 2.1% and 1.8%, respectively.

### Plasma XOR activity

Freshly frozen plasma samples maintained at − 80 °C were used to determine XOR activity with a method recently established for assays of stable isotope-labeled [^13^C_2_,^15^N_2_] xanthine with LC/TQMS at Mie Research Laboratories, Sanwa Kagaku Kenkyusho Co., Ltd, as we have previously described^8,9^. The intra- and inter-assay coefficients of variation of plasma XOR activity were 6.5% and 9.1%, respectively^8^.

### Statistical analysis

Data are expressed as number or median. Values for HOMA-IR, hs-CRP, and plasma XOR activity were logarithmically transformed before performing multivariable regression analyses, due to the skewed distribution. Multivariable regression analyses were performed to determine whether the obtained uric acid level was independently associated with BAP or d-ROMs level after adjustment with various clinical parameters, including plasma XOR activity, as well as age, gender, and smoking and alcohol habits, and VFA, SBP, total cholesterol, HbA1c, HOMA-IR, eGFR, and hs-CRP levels. The non-linearity of the effect of uric acid level on BAP or d-ROMs level was included in the regression model. A two-factor interaction term (gender * uric acid) was also incorporated into the multivariable regression analysis model to assess the effect of gender difference on the relationship of uric acid level with BAP as well as d-ROMs level. The R software package, version 3.6.3 (R Foundation for Statistical Computing, Vienna, Austria), and Statistical Package for the Social Sciences, version 22.0 (PASW Statistics), were used for data analysis. All reported *p* values are two-tailed, and a *p* value of < 0.20 was considered significant for non-linear and interaction effects, as noted in previous studies^52,53^, while a *p* value of < 0.05 was considered to indicate significance for all of the other analysis results.
